# Flexural Strength of 3D-Printing Resin Materials for Provisional Fixed Dental Prostheses

**DOI:** 10.3390/ma13183970

**Published:** 2020-09-08

**Authors:** Sang-Mo Park, Ji-Man Park, Seong-Kyun Kim, Seong-Joo Heo, Jai-Young Koak

**Affiliations:** 1 Department of Prosthodontics and Dental Research Institute, Seoul National University Dental Hospital, School of Dentistry, Seoul National University, 101 Daehak-ro, Jongno-gu, Seoul 03080, Korea; ryong028@hanmail.net (S.-M.P.); 0504heo@snu.ac.kr (S.-J.H.); young21c@snu.ac.kr (J.-Y.K.); 2 Department of Prosthodontics, Yonsei University College of Dentistry, 250 Seongsanno, Seodaemun-gu, Seoul 03722, Korea; jimarn@yuhs.ac

**Keywords:** 3D printing, additive manufacturing, flexural strength, three-unit resin prosthesis

## Abstract

The clinical application of 3D-printed provisional restorations is increasing due to expansion of intraoral scanners, easy dental computer-aided design (CAD) software, and improved 3D printing speed. This study compared flexural strength of 3D-printed three-unit fixed dental prostheses with that of conventionally fabricated and milled restorations. A metal jig of two abutments and pontic space and an indenter for flexural strength measurement were fabricated. A three-unit fixed dental prosthesis was designed and manufactured using three additive manufacturing technologies, with subtractive manufacturing and a conventional method as controls. Digital light processing (DLP) group specimens were prepared from a polymethyl methacrylate (PMMA)-based resin and printed with a DLP printer. Stereolithography (SLA) group specimens were prepared from PMMA-based resin and printed with an SLA printer, and fused deposition modeling (FDM) group specimens were from a polylactic acid-based resin and printed with an FDM printer. Flexural strength was investigated using a universal testing machine, and the results were statistically analyzed. DLP and SLA groups had significantly higher flexural strength than the conventional group (*p* < 0.001). No significant difference was observed in flexural strength between DLP and SLA groups. The FDM group showed only dents but no fracture. The results of this study suggest that provisional restorations fabricated by DLP and SLA technologies provide adequate flexural strength for dental use.

## 1. Introduction

Additive manufacturing (AM) technology reduces production process, time, and cost because it involves prototyping and producing custom-made parts in major industries such as automobiles, aviation, and machinery [1]. There is also the advantage of small-volume production of various types of consumer goods such as food, toys, and jewelry. In the medical field, AM technology is applied to patient-specific medical services, such as improving the accuracy and safety of surgery through surgical guides [2]. AM technology is also used for artificial organ transplantation by manufacturing artificial liver cells and artificial bronchial transplants using bioprinting. In the dental field, rapid prototyping skulls have been used for planning surgical procedures. Moreover, computer-guided implant surgical templates are produced for guided implant surgery [3,4]. Additionally, digital impressions can be acquired with an intraoral scanner and the data converted into an actual model or used to fabricate provisional restoration using a high-speed 3D printer on the day of tooth preparation. AM technology has been actively used in the dental field, such as in manufacturing complex partial denture frameworks and oral appliances that are less efficiently produced with milling.

After tooth preparation for a fixed dental prosthesis, a provisional restoration is manufactured to protect the dental pulp, restore periodontal health, and maintain occlusal relationships [2,3]. The manufacture of provisional restorations is possible with a direct method by cutting a ready-made crown, using a mold, or kneading a resin block. However, if the number of abutments increases, it is difficult to manufacture and takes a longer time. The indirect-direct method of replicating the abutment teeth of the study model on the computer-aided design (CAD) software, milling the polymethyl methacrylate (PMMA) resin disc to make a shell, and relining it after the abutment preparation, is effectively used in clinical practice. However, it is not easy to place the shell at the exact position in the case of a complete-arch restoration when there is limited information on adjacent teeth. Moreover, a pre-milled shell does not fit precisely when a retrofit crown is needed under the old removable partial denture. Therefore, the indirect method of producing provisional restoration by taking impressions immediately after tooth preparation may produce more satisfactory and precise results. However, making impressions, pouring the plaster, and manufacturing the restoration is a time-consuming process. With recent advances in intraoral scanners, chairside CAD, and high-speed 3D printers, the indirect method has become easy and quick to apply in clinical practice [5].

The use of AM for the fabrication of dental prostheses has many advantages compared to the use of a conventional (CV) method using impression materials and casting techniques such as the lost-wax technique. The AM method helps save materials and energy, reduces the carbon footprint, and is more economical than the CV method [4,5]. The subtractive manufacturing (SM) method shares many advantages of AM when compared to the CV method. However, the SM method is more wasteful than the AM method since the procedure entails cutting materials with a bur and produces heat, noise, and an unfavorable force. The AM method has considerable potential for application in dentistry. AM machines are now cheaper [4], smaller, and lighter than before [6]. They can work with various materials such as metals, ceramics, and polymers [2,7]. Recent studies have shown that dental prostheses manufactured using the AM method have an acceptable degree of precision compared to prostheses made using the SM and CV methods [1,8]. Furthermore, several studies have reported the application of AM in maxillofacial reconstruction [4,9,10,11,12], implant fixture construction and intervention [13,14], orthodontic appliances [15], metal bridges [6], guided tissue regeneration [16], tissue surface [17], and clasps of removable partial dentures [18].

Typical methods for printing polymer materials include fused deposition modeling (FDM), digital light processing (DLP), and stereolithography (SLA). In FDM, a liquefied filament is extruded from a nozzle and the material is fused on a scaffold when the nozzle is moved. The DLP method involves the polymerization of a photosensitive liquid resin in which a laser is controlled by a digital micro mirror. SLA is a method in which the same liquid resin is polymerized with a single laser beam. In the case of DLP, the entire layer of liquid resin is polymerized at once, making DLP faster than SLA [19]. The resolution of the DLP and SLA products is higher than that of the FDM. Hence, DLP and SLA can be used to fabricate delicate products with undercuts, such as dental devices. However, a liquid resin is somewhat difficult to handle, and SLA can be slower than FDM [20,21].

As 3D printing technology is used in dentistry, many related studies have been reported. One study reported that SLA-produced models were more accurate than the milled model [22]. Dimensional accuracy and surface roughness were measured in simplified crowns manufactured by FDM, DLP, multi jet fusion (MJF) and SLA printing [5]. The crown’s inner diameter was reduced, except for the DLP group, and the surface roughness of the FDM crown was the highest. Alharbi et al. manufactured a cylinder-shaped specimen using different printing directions and measured the compressive strength [23]. It was concluded that the specimen printed perpendicular to the direction in which the force was applied had high strength. Unkovskiy et al. calculated the precision accuracy for bar-shaped specimens [24]. These studies have only evaluated the physical properties or accuracy of a simplified form; few studies have evaluated forms that are frequently used in clinical practice.

The purpose of this study was to evaluate the effect of various AM principles on the flexural strength of three-unit provisional restorations. The null hypothesis was that the AM principles would not show any difference in flexural strength.

## 2. Materials and Methods 

### 2.1. Metal Jig and Indenter Production and Test Group Specimen Preparation

**Figure 1:**
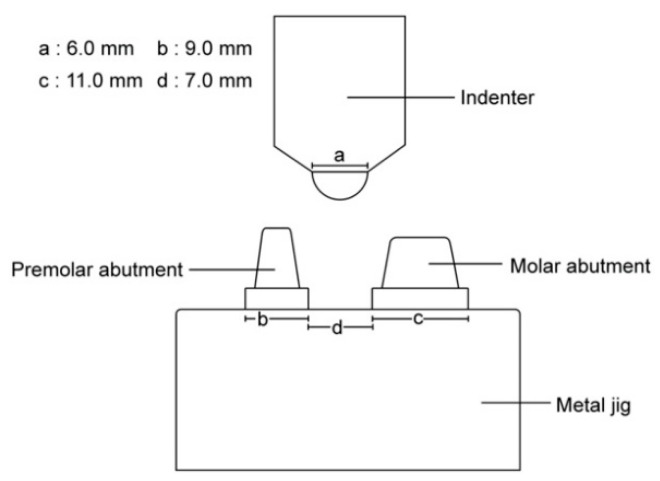
Schematic diagram of metal jig and indenter.

A metal jig, which was mounted on a universal test machine to hold the 3D-printed provisional restoration, was fabricated by adding two tapered and cylinder-shaped abutments. The indenter was manufactured to have a 6 mm diameter hemisphere for pressure application. Both were made of stainless steel by a milling procedure (Figure 1). 

**Figure 2:**
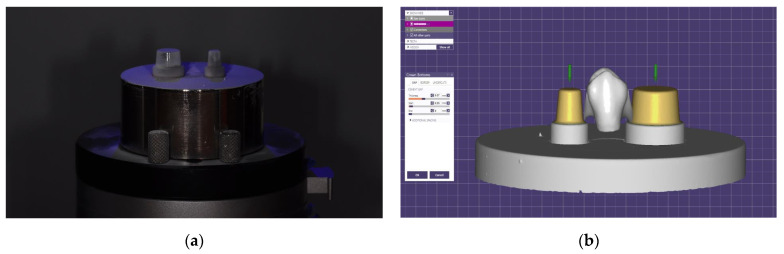

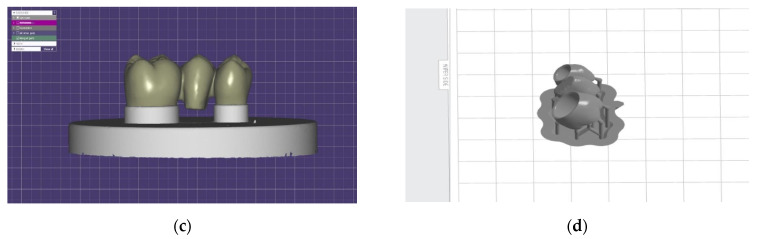
Design procedure for 3-unit restoration specimen. (**a**) Optical image of metal jig was acquired using a desktop scanner after scan powder was applied; (**b**) CAD parameters for fixed dental prosthesis were set in virtual model of the metal jig; (**c**) lateral view of completed design; (**d**) design data were loaded on pre-processing slicing software.

**Figure 3:**
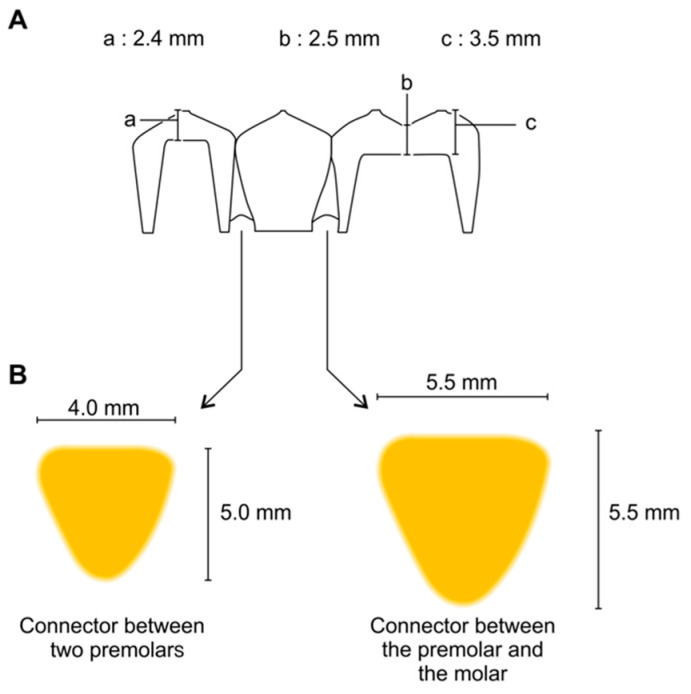
Design of 3-unit fixed dental prosthesis. (**A**) The dimensions of the specimen in the coronal section; (**B**) Cross section of the two connectors of the specimen.

For the flexural strength test, a 3-unit fixed dental prosthesis was designed on the metal jig and manufactured with 3D printers with various principles. Scan powder (Scan Spray; IP Division GmbH, Haimhausen, Germany) was applied to the metal jig to avoid the reflection of light during scanning. Then, the 3D image of the metal jig was acquired using a desktop scanner (T500, Medit Co., Seoul, Korea). The three-unit restoration was designed using dental CAD software (exoCAD; exoCAD, Darmstadt, Germany) (Figure 2). The connector between the premolar and the molar was 5.5 mm wide and 5.5 mm tall. The connector between the two premolars was 4 mm wide and 5 mm tall. In addition, the cross-section of the connecter was shaped like an inverted triangle with curved corners (Figure 3). Besides these dimensions, the thicknesses of the cusp, fossa, axial wall, and margins were considered when designing the connectors, according to the studies reported by Tinschert et al. [25]. The CAD parameters were set to provide 0.07 mm cement space 0.05 mm above the margin line for the restoration to be seated on the abutment. Completed design data were loaded on pre-processing software from each 3D printer manufacturer. 

The restorations were fabricated by three different 3D printing technologies, namely DLP, SLA, and FDM. For the first two groups, the PMMA-based liquid photopolymer resin material was poured into the vat of the 3D printer, and the specimens were printed according to the layer thickness recommended by each manufacturer (Table 1). For the FDM group, the specimens were built by heating up and extruding the thermoplastic filaments through the nozzle. The specifications of the materials for each group are presented in Table 2. The restoration design was positioned not to allow the support attachment to be connected to critical structures such as the margin or intaglio surface. For this purpose, the inner surface of the specimen was oriented upwards, and opposite to the base, and the building direction was set as 30° for all 3D printing groups. After the support attachment points were selected, the data were cut into slices according to the thickness recommended by each 3D printer manufacturer. DLP and SLA group specimens were immersed in 100% isopropyl alcohol to remove excessive resin monomers. The washed samples were post-cured using Denstar-300 (Denstar Co., Daegu, Korea). However, the FDM group did not undergo post-cure processing. While printing the specimens, the z-axis thickness was set to 100 µm for the DLP and SLA groups, and 200 µm for the FDM group. They were inspected to ensure a tight fit on the metal jig without rocking following the technique proposed by Naveen [26]. To determine the parameters for spacing, the specimens were adapted to the metal jig. When a specimen fitted firmly on the metal jig without locking, the particular spacing thickness used was considered appropriate. Fifteen specimens were obtained for each material.

### 2.2. Manufacturing Control Group Specimens

For the CV group, the specimens were prepared by pouring conventional PMMA-based self-cured resin (Jet Tooth Shade™ Powder; Lang Dental Co., Chicago, IL, USA). The tray was fabricated with a tray resin (Fstray; Bosworth Co., Midland, TX, USA). The mold was made by taking an impression of alginate irreversible hydrocolloid material (Aroma Fine Plus; GC Co., Tokyo, Japan). The monomer and the powder of the self-cured resin were mixed according to the manufacturer’s instructions. The resin mixture was inserted into the mold and seated on the metal jig for 20 min.

The SM group specimens were milled from a PMMA-based resin disc (ViPi block monocolor; VIPI Co., São Paulo, Brazil) using a milling machine (DWX-51; Roland DGA Cop., Irvine, KY, USA). The design data were imported into the CAM software (hyperDENT; FOLLOW-ME! TECHNOLOGY, Munich, Germany), and the tool path was calculated. Numerical data in the .nc format were copied into the milling machine control program, and the resin disc was processed.

### 2.3. Flexural Strength Measurements

The metal jig was fixed at the center of the universal testing machine (Instron 8871; Instron Co., Corwood, OH, USA). The rod with the indenter was placed at the top. The specimen was fitted on the abutment of the metal jig without cementation [26,27]. Pressure was applied with an increasing load of 10 kN at crosshead speed of 1 mm/min until the specimen fractured [28].

### 2.4. Patterns of Specimen Cracks and Fractures

The locations of cracks and fractures for each group specimen were investigated and divided into patterns. Morphological characteristics defined patterns. If the specimen was completely separated into fragments, the operator classified it as “F”. If the specimen was not completely divided and maintained its continuity, it was categorized as “C” for crack. When the specimen deformed without any fracture or crack, it was described as “D” for dent. 

### 2.5. Surface Evaluation of Printed Specimens

The specimens were observed with field emission scanning electron microscopy (FESEM) (Hitachi S-4700; Hitachi, Ltd., Tokyo, Japan).

### 2.6. Statistical Analysis

Statistical analysis of the flexural test from the materials was carried out using statistical software (SPSS; IBM Cop., New York, NY, USA). The normality of data distribution and homogeneity of variances were checked. The nonparametric Kruskal–Wallis and Mann–Whitney tests were used to analyze the data. The significance level was set at *p* < 0.05. 

## 3. Results

**Figure 4:**
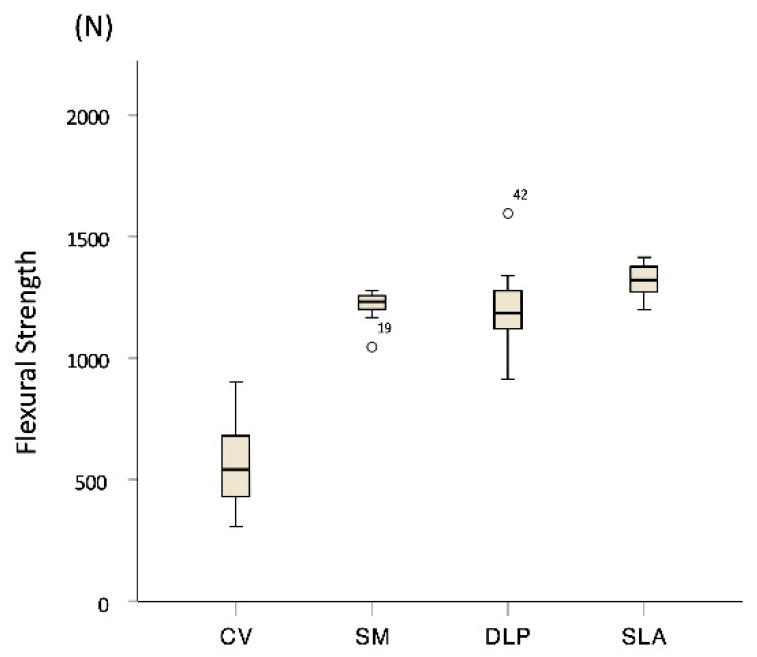
Flexural strength of each group. Different lowercase letters indicate statistical difference among groups (multiple comparison by Mann–Whitney U test with Bonferroni) (*p* < 0.05).

The medians and interquartile ranges (IQRs) of the flexural strength (N) for each group specimen, respectively, was 543 [IQR, 429–701] for CV, 1232 [IQR, 1193–1258] for SM, 1189 [IQR, 1110–1283] for DLP, and 1323 [IQR, 1245–1377] for SLA (Figure 4). No significant difference was observed between DLP and SM (*p* = 0.481). The SLA group had significantly higher flexural strengths than the other groups (*p* < 0.001), but no significant differences were observed between the DLP group and the positive control group SM (*p* > 0.05). The FDM group specimens did not break until the pontic contacted the metal jig’s base and were not fractured but only dented, making it impossible to collect data.

**Figure 5:**
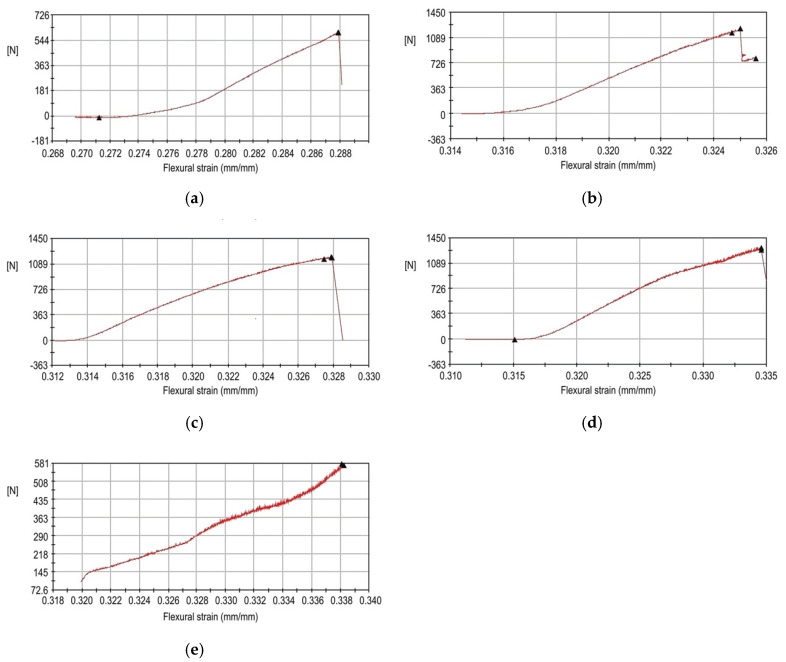
Flexural strain curves of restoration fabricated with different materials. (**a**) conventional (CV); (**b**) subtractive manufacturing (SM); (**c**) digital light processing (DLP); (**d**) stereolithography (SLA); (**e**) fused deposition modeling (FDM).

All of the specimen groups except FDM showed a sharp drop in force just before the breaking point in the flexural strain curves (Figure 5). The flexural strength of the FDM group did not show this drop in force, and the FDM group was excluded from the statistical analysis.

**Figure 6:**
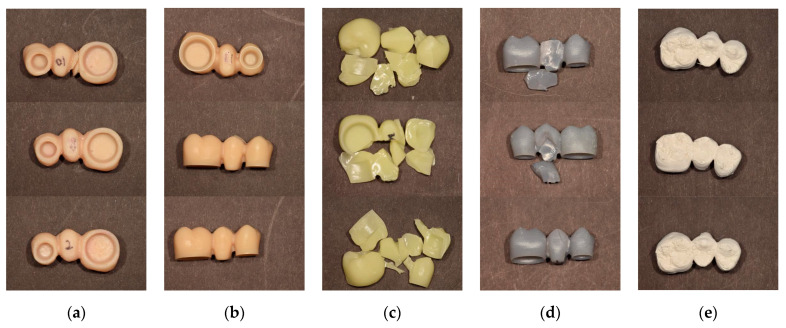
Fracture pattern of each specimen. (**a**) CV; (**b**) SM; (**c**) DLP; (**d**) SLA; (**e**) FDM.

When the location and shape of the cracks and fractures were evaluated, three significant patterns were observed. Morphological characteristics defined patterns. When the specimen was completely separated into fragments, ‘F’ was added after the initial fracture. If the specimen was not completely divided and maintained its continuity, “C” for crack was added. “D” for dent indicated the deformation without any fracture or crack. Concerning the range of the fracture, ‘a’ denotes a fracture or a crack over the entire area of the specimen. When the fracture or crack passes mainly on the pontic, “*p*” is added. “Fpa” denotes a pontic area fracture with small fragments, and “Fpb” indicates a connector fracture between pontic and retainer. Some specimens were dented with no fractures, others had cracks but no fractures, and some fractured into several pieces (Figure 6). Specimens in the CV group were completely divided into two fragments in the connector area and were classified as “Fpb”. The other specimens showed cracks in the pontic and connector area and were classified as “Cp”. The SM group specimens did not fracture but had cracks in the pontic and connector area and were classified as “Cp”. All of the DLP group specimens fractured into several fragments and were classified as “Fa”. Most of the SLA group specimens had small fractured fragments at the pontic area and were classified as ‘Fpa’. However, some specimens showed cracks just in the pontic area and were classified as “Cp”. The FDM group specimens did not show any fractures or cracks with the dent at the pontic occlusal surface and were classified as “Dp”. As a result, among the 15 specimens in the CV group, 12 restorations were cracked, while 3 were fractured. No SM group specimens were fractured but they were cracked. For the SLA group, 9 specimens were fractured, and 6 were cracked. All the SLA group specimens were fractured around the pontic area. All of the DLP group specimens were fractured into several pieces, not just around the pontic and connector area, but even at the crown (Table 3).

**Figure 7:**
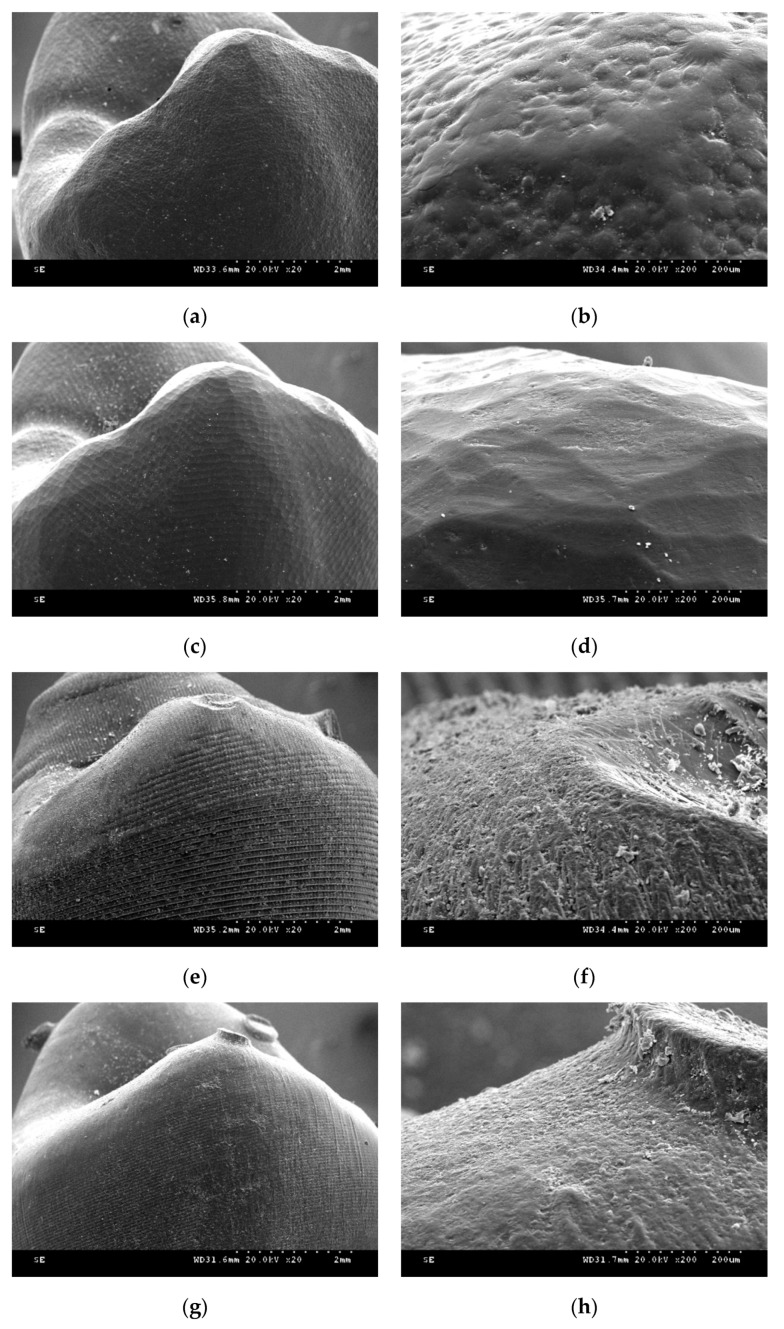

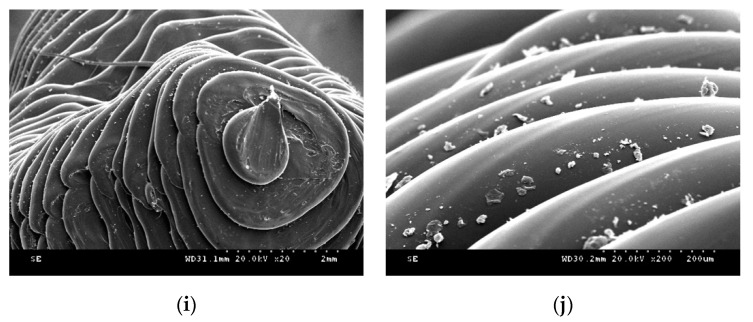
Scanning electron microscopy images after postprocessing procedure (oblique buccal view at buccal cusp tip of premolar). (**a**) CV (original magnification ×20); (**b**) CV (original magnification ×200); (**c**) SM (original magnification ×20); (**d**) SM (original magnification ×200); (**e**) DLP (original magnification ×20); (**f**) DLP (original magnification ×200); (**g**) SLA (original magnification ×20); (**h**) SLA (original magnification ×200); (**i**) FDM (original magnification ×20); (**j**) FDM (original magnification ×200).

Figure 7 shows FESEM images of the provisional restoration after each manufacture process. In self-cured resin specimens, small round grains were evenly distributed. The milled resin specimens showed homogeneous dented images. In the DLP printer group specimen, layer-to-layer transitions were stepped like a staircase. Additionally, the ×200 magnification image showed a rough surface, and the cured resin material flow was not flat. In the SLA group, the entire layer was clean, and the transition between the layers was well filled with the cured resin material. Each layer was thick in the FDM group, and the melted filaments were thinly stretched in the last layer.

## 4. Discussion

The flexural strength of the DLP and SLA groups was higher than the flexural strength of the negative control, the CV group. The flexural strength of the positive control, the SM group, did not differ significantly from that of the DLP group. Therefore, the null hypothesis was rejected. Furthermore, it is thought that 3D-printed products manufactured with DLP and SLA technologies can be used in clinical practice as the milled restoration from PMMA resin disk block can.

The FDM group showed considerably better flexibility than the other groups. However, the flexural strength of the FDM group specimen could not be conducted because the specimens were not fractured but deformed until the pontic base touched the base of the metal jig. Therefore, it was impossible to compare the flexural strength of the FDM group specimens with that of the other specimens. Different experimental conditions need to be considered for the fracture strength evaluation of restorations manufactured by the FDM method. The measured flexural strength of the CV group specimens produced in this study was not consistent with the manufacturer’s manual and was rather low. According to the manufacturer, the self-cured PMMA material of the CV group should be cured in a pressure pot (Aquapres; Lang Dental Co., Chicago, IL, USA) that can apply a hydraulic pressure of 30 psi for better results. However, the resin mixture was polymerized at normal pressure to simulate actual clinical conditions. If the abovementioned material was cured in the pressure pot at 30 psi, the flexural strength of the CV group might have been higher.

Although a lot of literature has been reported on 3D-printed dental products’ accuracy, there are not many references to mechanical properties. The dimensional accuracy of complete-arch model with fourteen prepared teeth printed with the SLA principle was reported to be better than the milled model [22]. When comparing additively manufactured complete-arch orthodontic models, the SLA group showed better trueness than FDM and DLP [29]. Alharbi et al. reported that the marginal gap and internal adaptation of single unit provisional restoration were better in the SLA 3D printing group than the milling group when evaluated by micro-CT imaging [30]. Oladapo et al. introduced a novel computational surface characterization for evaluating 3D-printed bone-implant and reported that tensile and compression stress was 102 and 29 MPa [31]. The flexural strength of bar-shaped specimen 3D printed from surgical template material with the SLA principle was 135–149 MPa [24]. When specimens were fabricated from provisional restoration material with SLA printer, the vertically printed cylinder’s compressive strength was 298 MPa, and that of the horizontally printed cylinder was 258 MPa [23]. The strength of 3D-printed dental devices varied depending on the shape and size of the sample. Although several studies comparing the strength of 3D-printed dental products have been reported, few pieces of literature compare the differences between various 3D printing principals. In this study, the flexural strength of 3-unit provisional restoration was highest in the SLA group. The reason for the SLA group’s high flexural strength can be found in the printed object’s surface morphology. In the DLP principle, each slice is cured of the single screen displayed from the DMD chipset, and the lines at each slice appear rough due to the chipset’s resolution limit. On the other hand, the SLA principal completes each slice as if drawing with a laser beam, so the object’s surface is relatively smooth. This can also be confirmed from the SEM photographs. At the specific area where the inter-layer bonding weak, the fracture can occur more quickly if the surface is rough.

In this study, we did not investigate the effect of cementation as in the studies of Naveen et al. [26] and Scherrer et al. [32], which reported that supports with a high elastic modulus increased the fracture toughness of the specimen. Rismanchian et al. also measured the flexural strength of an implant supported with zirconia without cementation [27]. Cementation increases the resistance of the fixed dental prosthesis against an external force by distributing the force evenly [33]. For this reason, test specimens were not cemented to the abutment teeth to evaluate the strength of the restoration itself. With regard to the accuracy, the optimal build direction for SLA printing was reported to be 120° by Alharbi et al. [34], while those for DLP printing was recommended to be 135° by Osman et al. [35]. The previous study confirmed that restoration 3D printed at 30°, 45°, and 60° have higher flexural strength than that manufactured at 0° and 90° [36]. Among the angulations of 30°, 45°, and 60°, printing at 30° resulted in the highest flexural strength value, and it is a condition in which the least amount of support is attached on the critical part and stably producing without failure. Because this study aims to compare each printing principal, the building direction was fixed at 30° to control other variables.

While the CV, SM, DLP, and SLA group specimens were fractured mainly around the connector and the pontic area, all of the FDM group specimens were only dented. This finding coincided with the results of studies that tested the flexural strength of three- or four-unit prostheses [25,27,33,37]. The retainer portion of the fixed dental prosthesis, i.e., just above the abutment teeth, did not fracture frequently. In this respect, it is important to achieve a strong connector structure when designing and manufacturing a three-unit prosthesis to lower the possibility of failure from fracture.

Moreover, there were various patterns of fractures or cracks in each group. The CV, SM, and SLA group specimens mostly had cracks, but the FDM group specimens showed no fractures and only dents under the experimental condition. Furthermore, the DLP specimens mostly fractured into several pieces. On the basis of these results, it can be said that the material used for the FDM group specimens had high elasticity, whereas those used for the DLP group specimens had significantly low elasticity. This is a characteristic of bisphenol acrylate-based acrylic materials that have good surface hardness but are brittle and derived from bisphenol’s chemical structure. The material used by the DLP group is presumed to have utilized this structure. On the other hand, the SLA group specimen’s fracture pattern was different, although it was produced with the same vat polymerization technique. It is presumed that urethane acrylate was used for the material of this group. Urethane acrylate is characteristically excellent in toughness but has a low surface hardness, a property derived from the urethane chemical bond. The SM group specimens did not fracture into sharp pieces, which shows that the resin used for the SM group specimens was safe against patient injury. In contrast, it is important to carefully check whether fractures occur, when fabricating provisional restorations with DLP technology in the clinical practice.

The FDM group showed the most characteristic change. This is because the material used in this group is PLA, while the basic component of the material used in the other groups is PMMA. The basic components of the material used in these groups are the same, but the fracture patterns of the CV, SM, DLP, and SLA specimens are diverse. Hence, it can be said that the properties of PMMA can vary according to the additional materials used in the different manufacturing methods. Hofstätter et al. [38] showed that the use of a fiber improved the tensile strength of DLP specimens. Gundrati et al. [39] showed that printing conditions affected the molecular alignment of the printed polymer. According to Hofstätter et al. [38], the use of a fiber interrupts crack propagation. The strength of a brittle material such as the photosensitive liquid resin of the DLP group in this study can be improved by adding a fiber. Thus, it is valuable to study the relationship between the fiber and the fracture pattern. How the fractures are created should be considered when printed prostheses are used in clinical practice. If the elasticity is too high, the fixation force of the prosthesis between the abutment teeth and the abutment decreases. In contrast, if the material elasticity is too low, the prosthesis will fracture into many pieces and may hurt the patient.

A limitation of this study was that it was an in vitro study. Additional studies should be conducted not only on the flexural strength but also on the fatigue strength along with solubility and permeability. Many factors determine the physical properties of a resin. Flexural strength may change when the resin specimen is surrounded by a solvent [40]. The nozzle temperature of FDM can affect the bonding strength of the filaments [41]. The printing speed and layer height also affect the tensile strength [42]. The use of fibers reinforces the tensile strength or wear resistance and contributes to anisotropic properties [38,43,44]. Many factors affect the permeability of a 3D polymer [39]. Therefore, the physical properties of a resin with respect to many factors should be studied in the future. Isopropyl alcohol was used to remove the resin monomer after printing in the DLP and SLA groups. However, this solvent significantly decreases the mechanical properties according to Väyrynen et al. [40]. Therefore, the effect of solvents such as isopropyl alcohol should be investigated. Other types of materials such as metals and ceramics are also used in clinics, but studies of the physical properties of these materials are scarcer than those of resin. Therefore, much needs to be explored in this area for safer and more versatile use of the AM technique.

## 5. Conclusions

Within the limitations of this in vitro study, the following conclusions were drawn. The experimental groups DLP and SLA had significantly higher flexural strengths than the CV negative control group. However, compared to the SM positive control group, no significant difference was observed in the flexural strength. The FDM group restoration was not fractured but dented, while the DLP group specimens fractured into several pieces. In the case of the CV and SLA groups, all of the specimens either had only cracks or cracks and fractures, while the SM specimens had only cracks.

## Figures and Tables

**Table 1:** Specifications of 3D printing devices.

Printer	Manufacturer	Build Volume	Layer Thickness	Spot Size	Power	Slicing Program	Post-Curing Time
DLP (D-150)	Nextdent Co.	150 mm × 84.3 mm × 100 mm	25–100 um	405 um (laser spot diameter)	150 W	VeltzBP	120 mins
SLA (Form2)	Formlabs Co.	145 mm × 175 mm × 175 mm	25–100 um	140 um (laser spot diameter)	65 W	PreForm	60 mins
FDM (Creator pro)	FlashForge Co.	227 mm × 148 mm × 150 mm	100–500 um	400 um (nozzle diameter)	350 W	Simplify3D	N/S

**Table 2:** Composition and mechanical properties of specimen materials.

Group	Manufacturing Method	Product Name	Manufacturer	Basic Materials
CV *	Self-curing	Jet Tooth ShadeTM Powder	Lang Dental Co.	Polymethyl methacrylate
SM *	Subtractive milling	ViPi	VIPI Co.	Polymethyl methacrylate
DLP **	Digital light processing	C&B	NextDent Co.	Polymethyl methacrylate
SLA **	Stereolithography	Standard (GPGR04)	Formlabs Co.	Poly Polymethylacrylate
FDM **	Fused deposit modeling	PLA	ColorFabb Co.	Polylactic acid

* Control groups ** Experimental groups.

**Table 3:** Classification of characteristic patterns of cracks and fractures.

Specimen number	CV	SM	DLP	SLA	FDM
1	Cp	Cp	Fa	Fpa	Dp
2	Cp	Cp	Fa	Fpa	Dp
3	Cp	Cp	Fa	Fpa	Dp
4	Cp	Cp	Fa	Cp	Dp
5	Cp	Cp	Fa	Cp	Dp
6	Fpb	Cp	Fa	Cp	Dp
7	Fpb	Cp	Fa	Fpa	Dp
8	Cp	Cp	Fa	Fpa	Dp
9	Cp	Cp	Fa	Cp	Dp
10	Fpb	Cp	Fa	Cp	Dp
11	Cp	Cp	Fa	Fpa	Dp
12	Cp	Cp	Fa	Fpa	Dp
13	Cp	Cp	Fa	Fpa	Dp
14	Cp	Cp	Fa	Cp	Dp
15	Cp	Cp	Fa	Fpa	Dp

“F” for fracture: completely separated into fragments; “C” for crack: not completely divided and maintained its continuity; “D” for dent: deformation without any fracture or crack; “a” designates a fracture or a crack passed over the entire area of the specimen; “p” designates a fracture or crack passed mainly on the pontic; “Fpa” denotes a pontic area fracture with small fragments; ‘Fpb’ indicates a connector fracture between pontic and retainer.
